# Comparative clinical genetic testing in spontaneous miscarriage: Insights from a study in Southern Chinese women

**DOI:** 10.1111/jcmm.16588

**Published:** 2021-05-10

**Authors:** Meiying Cai, Na Lin, Liangpu Xu, Hailong Huang

**Affiliations:** ^1^ Department of the Prenatal Diagnosis Center Fujian Maternity and Child Health Hospital Fujian Key Laboratory for Prenatal Diagnosis and Birth Defect Affiliated Hospital of Fujian Medical University Fuzhou China

**Keywords:** genetic abnormalities, single nucleotide polymorphism array, spontaneous miscarriages

## Abstract

Single nucleotide polymorphism (SNP) array and karyotype analyses were conducted on 441 spontaneous miscarriage placental villous tissues collected from women from southern China. Subsequently, the results from these two analyses were compared to evaluate the best diagnostic strategy for subsequent pre‐pregnancy planning. Here, the success rate of genetic testing using karyotyping and SNP array analysis was 78.46% (346/441) and 100.0% (441/441), respectively. The abnormality rate estimated by both methods was 54.9% (242/441). Three hundred and forty‐six cases were successfully detected via both SNP array and karyotype analyses; the rate of consistent detection was 96.24% (333/346), whereas 13 cases were not consistent. There was no substantial positive correlation between age and genetic abnormalities such as Turner syndrome, structural variation or euploidy state in the different age groups studied. However, the aneuploidy rate was significantly different in each age group. Thus, although SNP array has higher success rate and resolution in genetic abnormality detection, supplementary karyotype analysis is needed for a more accurate revelation of the genetic aetiology of miscarriages. Therefore, this study indicates that simultaneous karyotype and SNP array analyses should be performed for spontaneous miscarriages. Furthermore, miscarriages irrespective of maternal age must be genetically analysed.

## 1 INTRODUCTION

Spontaneous miscarriage is a common complication of pregnancy, accounting for approximately 6%–14% of clinical pregnancies in China.^1^, ^2^, ^3^, ^4^ Its aetiology is very complex, mainly including genetic, immune and endocrine factors; uterine lesions; and female reproductive tract infection.^5^, ^6^, ^7^ Approximately, 45%~60% of spontaneous miscarriages are caused by chromosomal abnormalities.^8^, ^9^, ^10^, ^11^ Genetic analysis of abortion villous tissues is a necessary method to detect the genetic causes of foetal loss and accurately assess the risk at subsequent pregnancy recurrence.^11^, ^12^, ^13^ Traditional genetic analysis involves cell culture and G‐banding karyotype analysis, but this method is limited by the success rates of culture and resolution. In recent years, chromosome microarray analysis (CMA), also known as ‘molecular karyotype analysis’, has been gradually applied to the genetic analysis of abortion villous tissues due to advantages, such as no requirement for culture and high throughput and high resolution.^14^, ^15^, ^16^, ^17^, ^18^ CMA can be divided into two categories: array‐based comparative genomic hybridization ^19^, ^20^, ^21^ and single nucleotide polymorphism (SNP) array.^22^, ^23^ In addition to detecting copy number variation (CNV), SNP array analysis can also detect loss of heterozygosity (LOH), uniparental disomy, triploidy^15^ and a certain level of mosaicism.^24^

Here, we investigated the feasibility and superiority of the two methods, traditional karyotyping vs. SNP array analysis, for genetic analysis of abortion villous tissues in 441 cases of spontaneous miscarriages in southern China.

## 2 MATERIALS AND METHODS

### 2.1 Study Participants and Samples

Abortion villous tissues were collected from 441 women with spontaneous miscarriage between November 2016 and September 2020 (Fujian Maternal and Child Health Hospital, China). The participating women (participants) ranged in age from 19 to 47 years, with an average age of 31.7 years. The gestational age of the participants ranged from 6 to 13 weeks, with an average gestational age of 8.1 weeks. The participants were divided into four groups based on age: < 30‐year‐old group; 30‐ to 34‐year‐old group; 35‐ to 39‐year‐old group; and ≥40‐year‐old group. Peripheral blood from both pregnant women participants and their spouses (participating couples) were also collected to exclude maternal cell contamination and to assist in interpretation of the test results where necessary. All samples were obtained with the informed consent of the pregnant women and their family members. The study was approved by the Ethics Committee of the Fujian Provincial Maternity and Children's Hospital. Written informed consent was obtained from all participants.

### 2.2 Villous cell culture and traditional karyotyping

Under aseptic conditions, the villous tissues were rinsed with 0.9% sodium chloride solution and separated from blood clots and non‐villi tissue; subsequently, high‐quality villi were selected. The selected villi were chopped and divided into two parts, 15‐25 mg each for cell culture and DNA extraction, respectively. The villous tissues selected for cell culture were inoculated, cultured and sectioned according to the conventional method. After banding, the slides were placed on a GSL‐120 automatic chromosome scanner for scanning. Five karyotypes from 20 mitotic phases were analysed following the ISCN 2013 standards. If mosaicism occurred, 10 karyotypes from 40 the mitotic phases were analysed.

### 2.3 SNP array analysis

Genomic DNA was extracted using the Qiagen kit (Germany), and the concentration and purity of DNA were determined via ultraspectrophotometry. DNA digestion, amplification, purification, fragmentation, labelling, hybridization, washing, staining and scanning were performed using Affymetrix CytoScan 750K microarray (USA). Results were analysed using the matching Chromosome Analysis Suite V3.2, and SNP array results were further analysed in combination with relevant databases to determine the nature of CNV. The referenced databases include DGV (http://dgv.tcag.ca/dgv/app/home), OMIM (https://www.omim.org/), DECIPHER(https://decipher.sanger.ac.uk/index) and PubMed (https://www.ncbi.nlm.nih.gov/). The results were divided into three categories based on the nature of CNVs: pathogenic CNVs, benign CNVs and uncertain clinical significance (VUS) CNVs.^25^ To avoid maternal cell contamination during cordocentesis, short‐tandem repeats analysis was applied before testing. Maternal blood sample and miscarriage placental villous DNA were analysed in parallel to detect MCC.

### 2.4 Statistical analysis

SPSS Statistics v20 software (IBM, Armonk, NY) was used. Statistical comparisons with groups were performed using chi‐square test, and a P‐value of <0.05 was considered statistically significant.

## 3 RESULTS

### 3.1 Traditional karyotyping is effective but plagued by culture success and karyotype quality

Three hundred and forty‐six of the 441 abortion villous tissues could be karyotyped successfully, whereas 95 could not be karyotyped. Thus, the karyotyping success rate was 78.46% (346/441). Among the 95 failed cases, in 75 cases, analysable chromosomes could not be obtained after fluid exchange and passage treatment due the slow growth of cell clones in villi as the embryonic development had ceased for long time. The other 20 cases could not be karyotyped because the cultures failed due to contamination.

Among the 346 cases with successful cell culture and karyotyping, 186 cases (53.76%, 186/346) had abnormal karyotypes of which 179 cases (96.24%, 179/186) showed abnormal chromosome numbers, mostly trisomy. Seven cases (3.76%, 7/186) had abnormal chromosomal structure (Table 1).

**TABLE 1:** The genetic abnormalities detected via traditional karyotyping and SNP array

Type	SNP array (n = 441)	Karyotype analysis (n = 346)
Number abnormality	215	179
Trisomy	162	132
Turner syndrome	23	21
Mosaicism	11	9
Triploid	9	8
Tetraploid	0	5
Double trisomy	10	4
Structural abnormality	20	7
Normal karyotype	206	167

### 3.2 SNP array analysis shows higher genetic testing success rate

All 441 villous tissues could be analysed via SNP array. Thus, the success rate was 100% (441/441). SNP array analysis was able to detect 47 cases of chromosomal abnormalities among the 95 karyotyping failure samples. Forty‐two of these cases were aneuploidies, whereas five cases were structural abnormalities. Thus, a total of 235 cases of chromosomal abnormalities were detected via SNP array analysis, and the abnormality rate was 53.29% (235/441). Among 235 cases of chromosomal abnormalities, 215 cases (91.49%) were aneuploidies, including 162 cases of trisomy, 23 cases of Turner syndrome, 11 cases of mosaicism, 10 cases of double trisomy and nine cases of triploidy. Moreover, 8.51% (20/235) of the detected abnormalities were structural (Table 1).

**FIGURE 1:**
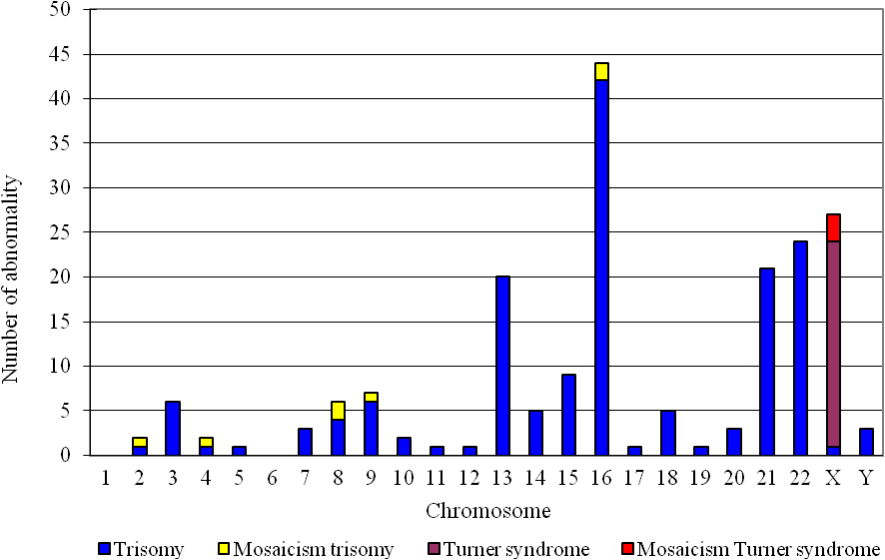
Distribution of single chromosome aneuploidy among the different human chromosomes

There were 195 cases of single chromosome aneuploidy (including 11 cases of mosaicism) (Figure 1). Moreover, this single chromosome aneuploidy was seen to affect most chromosomes, except chromosomes 1 and 6. The incidence of chromosome 16 trisomy was the highest (44/195, 22.56%, including two mosaicisms), followed by 24 cases of trisomy 22, 21 cases of trisomy 21 and 20 cases of trisomy 13, respectively. Chromatids mainly occurred on the X chromosome (a total of 27, including three mosaicisms).

Twenty cases with structure abnormalities were detected via SNP array analysis. However, the parental couples of the two LOH cases detected via SNP array analysis refused peripheral blood verification tests (Table 2). Thus, peripheral blood karyotype analysis for structural abnormalities was carried out in 18 parents of the 20 spontaneous miscarriages; structural abnormalities were detected via villous SNP array. Two maternal carriers of balanced chromosomal translocation and two maternal carriers of inverted chromosomal inversion were found, whereas the remaining 14 parental couples had normal karyotype.

**TABLE 2:** The parental peripheral blood karyotyping results of the 20 cases in which abortion villous tissue SNP array revealed chromosomal structural anomalies

Case	SNP array	Pathogenicity classification	Paternal karyotype	Maternal karyotype
1	arr[hg19]1p36.33p36.32(849,466‐2,579,267) x3,15q26.1q26.3(94,233,409‐102,429,040) x1	P	46,XY	46,XX,t(1;15)(p36.3;q26.1)
2	arr[hg19]6p25.3q13(294,825‐75,334,384) x3	P	46,XY	46,XX,t(6;15)(q13;p11.2)
3	arr[hg19]7q11.23(74,175,031‐74,566,129)x1,10q11.22q11.23(49,730,919‐50,395,827)x3, 14q23.2(63,970,519‐64,284,284)x1, Yp11.2(7,643,381‐8,808,561)x2	P	46,XY	46,XX,inv(7)(q31.3q22)
4	arr[hg19]4p16.3p16.1(68,345,‐8,721,580) x1,4q11q35.2(52,686,030‐190,957,460) x3	P	46,XY	46,XX,inv(4)(p16.1q12)
5	arr[hg19]2q32.1q32.2(189,194,304‐190,487,242)x3, Xp22.12p11.21(21,782,384‐56,905,943)x1, Xq12q28(65,783,010‐155,160,723)x1	P	46,XY	46,XX
6	arr[hg19]14q11.2q32.33(20,516,277‐107,284,437)x3,21q11.2q22.3(15,016,486‐48,093,361)x1	P	46,XY	46,XX
7	arr[hg19]8p23.3p11.22(158,048‐38,587,551)x1,22q13.31q13.33(46,849,180‐51,072,556)x3	P	46,XY	46,XX
8	arr[hg19]10q25.1q26.3(106,089,381‐132,870,670)x2‐3,10q25.2q25.3(114,235,295‐115,41,953) x3,10q26.6q26.3(130,066,717‐132,733,665) x3,10q26.3(133,858,562‐135,426,386) x1	P	46,XY	46,XX
9	arr[hg19]8p23.3p23.1(158,048‐11,935,465)x1,8p22p12(12,786,593‐30,386,265)x1‐2,14q31.1q32.33(80,773,607‐107,284,437)x2‐3	P	46,XY	46,XX
10	arr[hg19]22q13.31q13.33(44,261,580‐51,197,766)x1	P	46,XY	46,XX
11	arr[hg19]7q31.2q34(115,729,160‐141,679,588)x3, 7q34q36.3(141,687,274‐159,119,707)x1	P	46,XY	46,XX
12	arr[hg19]8p23.3p11.21(158,048‐469,480) x1,20p13p12.1(61,661‐15,916,956) x3	P	46,XY	46,XX
13	arr[hg19]Xp22.33q28(168,551‐154,669,330)x1‐2	P	46,XY	46,XX
14	arr[hg19]Yp11.31p11.2(2,650,424‐6,356,292) x0, Yp11.2(7,251,143‐9,745,027) x0	P	46,XY	46,XX
15	arr[hg19]5p15.33p15.2(113,576‐14,921,416) x3,11q24.1q25(122,084,943‐134,529,443) x1	P	46,XY	46,XX
16	arr[hg19]8p23.3p12(158,048‐33,547,773) x1	P	46,XY	46,XX
17	arr[hg19]7q34q36.3(142,342,270‐159,119,707) x1,8q22.3q24.3(106,063,542‐146,295,771) x3	P	46,XY	46,XX
18	arr[hg19]16p11.2(29,696,959‐30,165,725) x3	P	46,XY	46,XX
19	arr[hg19]13q11q21.31(19,450,956‐63,383,496) hmz,13q21.33q34(73,111,757‐115,095,705)hmz	VUS	‐	‐
20	arr[hg19]6q21q23.3(109,019,605‐136,245,611) hmz,14q13.1q23.2(34,585,230‐62,540,298) hmz	VUS	‐	‐

Abbreviations: P, pathogenic; VUS, uncertain clinical significance.

### 3.3 Genetic testing of spontaneous miscarriage villous tissue with traditional karyotyping and SNP array analysis: Comparison reveals both methods effective but supplement each other

A total of 441 cases were analysed via karyotyping and SNP array analysis. The detection success rate of SNP array analysis (100%, 441/441) was higher than that of karyotyping (78.46%, 346/441). The difference in the abnormality rates detected by the two methods was statistically not significant (*P* >.05), 53.29% (235/441) and 53.76% (186/346), for SNP array analysis and karyotyping, respectively. Of the 346 cases analysed successfully via both methods, abnormalities detected were consistent in 333 cases and inconsistent in 13. Thus, the consistency rate was 96.24% (333/346). The abnormalities detected in 13 cases which showed inconsistent results via both methods are as follows: five cases of tetraploidy, four cases of microduplication and microdeletion, two cases of LOH, one case of abnormal balance structure and one case of low proportion mosaicism of X chromosome. Moreover, the cases with tetraploidy, equilibrium structure abnormality and low proportion mosaicism of X chromosome could not be detected via SNP array analysis. However, karyotyping could not detect microduplication, microdeletion and LOH (Table 3).

**TABLE 3:** The inconsistent results by karyotype analysis and SNP array

Case	Karyotype analysis	SNP array
1	92,XXYY	Normal
2	92,XXYY	Normal
3	92,XXYY	Normal
4	92,XXYY	Normal
5	92,XXYY	Normal
6	45,XX,rob(13;14)(q10;q10)	Normal
7	45,X[11]/46,XY[29]	Normal
8	Normal	arr[hg19]Yp11.31p11.2(2,650,424‐6,356,292) x0, Yp11.2(7,251,143‐9,745,027) x0
9	Normal	arr[hg19]1p36.33p36.32(849,466‐2,579,267)x3,15q26.1q26.3(94,233,409‐102,429,040) x1
10	Normal	arr[hg19]16p11.2(29,696,959‐30,165,725) x3
11	Normal	arr[hg19]22q13.31q13.33(44,261,580‐51,197,766)x1
12	Normal	arr[hg19]6q21q23.3(109,019,605‐136,245,611)hmz,14q13.1q23.2(34,585,230‐62,540,298) hmz
13	Normal	arr[hg19]13q11q21.31(19,450,956‐63,383,496)hmz,13q21.33q34(73,111,757‐115,095,705) hmz

### 3.4 Chromosomal abnormalities seen in abortion villous tissues independent of maternal age, whereas aneuploidy frequency differs with maternal age

**FIGURE 2:**
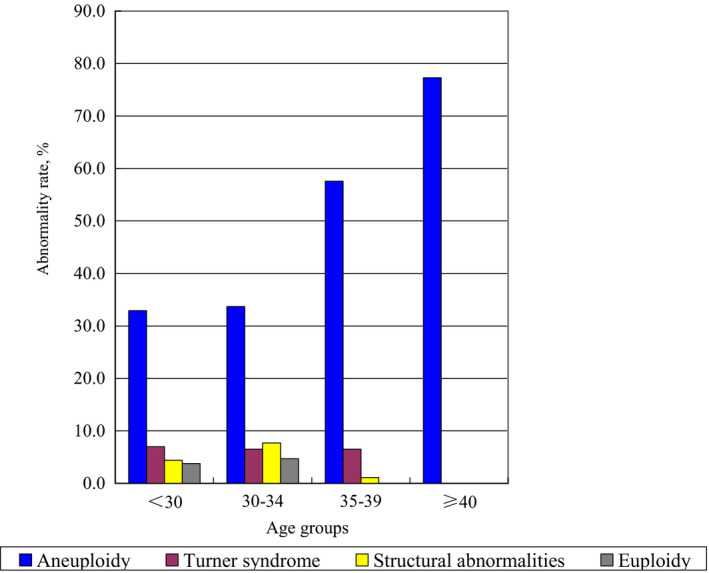
Distribution of aneuploidy, Turner syndrome, structural abnormalities and euploidy in different maternal age groups

A total of 441 cases were analysed by karyotyping and SNP array analysis, and 242 cases with genetic abnormalities were detected, including 179 cases of aneuploidy (trisomy and two number abnormalities), 28 cases of Turner syndrome, 21 cases of structural abnormalities and 14 cases of euploidy (triploidy and tetraploidy) (Figure 2). The frequency of these four abnormal types (aneuploidy, Turner syndrome, structural abnormalities, euploidy) in different age groups (< 30‐year‐old group, 30‐ to 34‐year‐old group, 35‐ to 39‐year‐old group, and ≥40‐year‐old group) was different (Table 4). Statistical analysis showed that there was no significant positive correlation between age and the different chromosomal abnormalities such as Turner syndrome, structural abnormalities and euploidy (*P* >.05). However, the aneuploidy abnormality rate in 35‐ to 39‐year‐old group and ≥40‐year‐old group was significantly higher than that in <30‐year‐old group and 30‐ to 34‐year‐old group (*P* <.05). The aneuploidy abnormality rate was the highest in ≥40‐year‐old group, followed by the 35‐ to 39‐year‐old group.

**TABLE 4:** The frequency of the four abnormality types (aneuploidy, Turner syndrome, structural abnormalities and euploidy) in different maternal age groups

Age group	Total	Aneuploidy (Abnormality rate, %)	Turner syndrome (Abnormality rate, %)	Structural abnormality (Abnormality rate, %)	Euploidy (Abnormality rate, %)
<30	158	52(32.9)*	11(7.0)	7(4.4)	6(3.8)
30‐34	169	57(33.7)*	11(6.5)	13(7.7)	8(4.7)
35‐39	92	53(57.6)*	6(6.5)	1(1.1)	0(0)
≥40	22	17(77.3)*	0(0)	0(0)	0(0)

* *P* <.05 compared with group via chi‐square test.

## 4 DISCUSSION

The aetiology of spontaneous miscarriage is complex, and chromosomal abnormality is the main cause.^26^, ^27^, ^28^ Chromosomal analysis of placental villi in early abortion can clarify the foetal loss caused by chromosomal abnormalities.^2^, ^13^, ^22^, ^29^ Traditional karyotyping is the ‘gold standard’ of cytogenetic diagnosis. It can detect aneuploidy and microscopically visible structural abnormalities. However, failure of cell culture and poor karyotypic morphology may affect its success rate and accuracy. The failure rate of karyotyping in detection of abnormalities in abortion villus tissue is as high as 40%.^30^, ^31^ Here, the success rate of karyotyping was 78.46% (346/441). SNP array is a recently developed molecular genetics technique. Here, all 441 villous tissues could be analysed by SNP array, and thus, the success rate was 100% (441/441). Additionally, SNP array analysis could detect 47 cases of chromosomal abnormalities in the 95 cases in which karyotyping failed. Forty‐two of these cases were number abnormalities, and five cases were structural abnormalities. Therefore, SNP array has a significant advantage in cases where karyotyping fails. In addition, SNP array has higher resolution and can detect microdeletions or microduplications; such abnormalities cannot be detected by karyotyping at the genome level.^19^, ^32^, ^33^, ^34^

Furthermore, here, a total of 20 cases with structural abnormalities were detected by SNP array analysis. Eighteen of these cases were pathogenic CNVs, and two were LOH. Karyotyping was carried out in the parental peripheral blood of these 18 aborted embryos with structural abnormalities. Results revealed two cases of maternal balanced translocation carriers and two cases of maternal inverted chromosome carriers, indicating that the structural abnormalities seen in aborted embryo villi were maternally inherited. The parental karyotyping of the remainder 14 aborted embryos did not show any genetic abnormality. Thus, the embryonic structural abnormalities may be formed de novo or may reflect the resolution errors inherent in traditional G‐banding karyotyping and SNP array analysis; small fragment balanced translocations are tough to detect. Thus, these couples with history of spontaneous miscarriage showing no genetic abnormalities will be monitored carefully for understanding their future pregnancy outcomes as a continuation of this study. The two LOH cases detected by SNP array analysis were declared VUS as the genetic cause of these spontaneous miscarriage was not clear because the couples refused peripheral blood genetic analysis. Thus, guidance for next pregnancy cannot be given in the absence of knowledge of genetic abnormality underpinning the miscarriage.

Approximately half of early spontaneous miscarriage is caused by chromosome abnormality.^35^ Here, a total of 441 cases were analysed by karyotyping and SNP array analysis, and the abnormality detection rate was 54.9% (242 /441). This is consistent with the results of previous studies.^20^ Abnormal chromosome number is the most important genetic factor that causes spontaneous miscarriage. Here, 221 cases (91.3%, 221/242) were number abnormalities. These number abnormalities included 195 cases of single chromosome aneuploidy (incidence distributed over all chromosomes excepting chromosomes 1 and 6). The incidence of chromosome 16 trisomy was the highest (22.56%, 44/195), followed by trisomy 22 and trisomy 21. This is consistent with previous research reports.^33^, ^36^ In trisomy, the gene dosage effect inhibits development and survival of zygote resulting in spontaneous miscarriage or embryonic death. Aneuploidy is caused by non‐separation of homologous chromosomes during meiotic formation of germ cells, leading to the chromosomal number abnormality in the zygote.

Among the 13 non‐overlapping cases, karyotyping failed to detect the four cases of microduplication and microdeletion, and two cases of LOH detected by SNP array method. This may be due to the difficulty in achieving good resolution caused by the unsatisfactory banding of villi chromosomes during karyotyping. Thus, SNP array analysis can detect LOH, microduplications and microdeletions that karyotyping cannot detect. Therefore, SNP array analysis helps in finding the cause of spontaneous miscarriage and provides a basis for subsequent prenatal or pre‐implantation diagnosis or screening.

However, the tetraploidy, equilibrium structure abnormality and low proportion mosaicism of X chromosomes (which also comprise the 13 non‐overlapping cases) could not be detected by SNP array analysis. Thus, karyotyping has an irreplaceable advantage in the detection of such types of abnormalities.

In summary, the results of this study indicate that a variety of chromosomal abnormalities lead to spontaneous miscarriage. Therefore, SNP array analysis cannot completely replace karyotyping. Moreover, the vast information output of SNP array analysis requires intensive interpretation abilities.

The risk of miscarriage due to chromosomal abnormalities is increased in women over 35 years of age.^37^, ^38^, ^39^ In brief, the older the female is, the more likely the embryo is to have aneuploidy, most probably due to the gradual degeneration of ovarian function with age which may cause the chromosomes to not separate well during the formation of germ cells.^6^, ^13^, ^40^ However, the incidence of Turner syndrome, structural abnormalities and euploidy was not directly related to the maternal age. The results of this study will be revised continually with the availability of new clinical data from our ongoing research to reflect more truly the relationship between various chromosomal abnormalities and maternal ages. Nevertheless, from this study, it can be inferred that in cases of spontaneous miscarriages, genetic testing of abortion villous tissues is recommended for aetiology analysis irrespective of the maternal age. This study is the first to formulate this view.

The shortcoming of this study is lack of accuracy in judging the occurrence of real mosaicism. In addition, relationship between frequency of spontaneous miscarriage and chromosomal abnormality has not been evaluated.

Various chromosomal abnormalities lead to spontaneous miscarriage. To identify the cause of spontaneous miscarriage and to provide evidence for risk assessment of the next pregnancy, we recommend that patients undergo both tests. Where this is not feasible, karyotyping may be recommended first, followed by SNP array analysis in cases where karyotyping is not possible due to villous cell culture failure or karyotyping results did not reveal any abnormalities. Furthermore, genetic testing is recommended for spontaneous miscarriages irrespective of maternal age. By detecting the chromosomal abnormalities in the aborted villous tissue, pre‐pregnancy planning may be strategized for avoiding miscarriage recurrence. Thus, providing a scientific and accurate molecular genetic diagnosis basis is essential for targeted pre‐pregnancy eugenic measures in the next pregnancy.

## CONFLICTS OF INTEREST

The authors declare no conflicts of interest.

## AUTHOR CONTRIBUTION

**Meiying Cai:** Writing‐original draft (equal). **Na Lin:** Project administration (equal). **liangpu xu:** Conceptualization (equal). **Hailong Huang:** Writing‐review & editing (equal).

## Data Availability

All data generated during and/or analysed during the current study are available upon request by contact the corresponding author.
